# Discovery of small molecule inhibitors of MyD88-dependent signaling pathways using a computational screen

**DOI:** 10.1038/srep14246

**Published:** 2015-09-18

**Authors:** Mark A. Olson, Michael S. Lee, Teri L. Kissner, Shahabuddin Alam, David S. Waugh, Kamal U. Saikh

**Affiliations:** 1 Department of Cell Biology and Biochemistry and; 2 Department of Immunology, Molecular and Translational Sciences Division, U.S. Army Medical Research Institute of Infectious Diseases, Frederick, MD 21702; 3 Macromolecular Crystallography Laboratory, National Cancer Institute at Frederick, Frederick, MD 21702; 4 Computational Sciences Division, U.S. Army Research Laboratory, Aberdeen Proving Ground, MD 21005.

## Abstract

In this study, we used high-throughput computational screening to discover drug-like inhibitors of the host MyD88 protein-protein signaling interaction implicated in the potentially lethal immune response associated with *Staphylococcal* enterotoxins. We built a protein-protein dimeric docking model of the Toll-interleukin receptor (TIR)-domain of MyD88 and identified a binding site for docking small molecules. Computational screening of 5 million drug-like compounds led to testing of 30 small molecules; one of these molecules inhibits the TIR-TIR domain interaction and attenuates pro-inflammatory cytokine production in human primary cell cultures. Compounds chemically similar to this hit from the PubChem database were observed to be more potent with improved drug-like properties. Most of these 2^nd^ generation compounds inhibit *Staphylococcal* enterotoxin B (SEB)-induced TNF-α, IFN-γ, IL-6, and IL-1β production at 2–10 μM in human primary cells. Biochemical analysis and a cell-based reporter assay revealed that the most promising compound, T6167923, disrupts MyD88 homodimeric formation, which is critical for its signaling function. Furthermore, we observed that administration of a single dose of T6167923 completely protects mice from lethal SEB-induced toxic shock. In summary, our *in silico* approach has identified anti-inflammatory inhibitors against *in vitro* and *in vivo* toxin exposure with promise to treat other MyD88-related pro-inflammatory diseases.

Among the vast network of cellular protein-protein interactions, a key trigger of innate immune signaling is the interactions predominantly mediated by Myeloid differentiation primary response protein 88 (MyD88). MyD88 is a 31–33 kDa adaptor protein containing an N-terminal death domain (DD) and a C-terminal Toll/interleukin-1 receptor (TLR/IL-1R) (TIR) domain separated by a short linker region^1^. The protein functions as an anchor to recruit signaling proteins to the (TLR/IL-1R) receptors^2^,^3^, as well as an IFN-γ receptor^4^ associated with induction of innate immune response. In addition, MHC class II molecules, which also serve as cell-surface receptors, engage MyD88 for innate immune signaling^5^,^6^,^7^. MyD88 performs this act with delicate precision. Perturbed regulation or excessive stimulation of the innate immune system *via* MyD88 may trigger amplification of the inflammatory signaling that can spiral out of control, leading to a profound clinical syndrome^8^ and severe pathological consequences, including toxic shock, sepsis and death^9^,^10^. The crucial role of MyD88 in these disorders suggests it is an important target for therapeutic intervention in limiting undesirable immune responses; namely, immune-pathogenesis. In fact, recent experimental results from our laboratory demonstrated that MyD88 gene knockout mice are protected from toxic shock-induced death upon lethal challenge of *Staphylococcal* enterotoxin B (SEB) with significantly reduced levels of pro-inflammatory cytokines in their serum^10^. In contrast, the potent cytokine response of wild-type mice was significant and lethal^10^. In addition to toxin exposure, other studies suggest that MyD88 activation plays a critical role in immune pathogenesis as well^11^.

The TIR domains of adaptor proteins like MyD88 mediate the initial events that occur after pathogen recognition by toll-like receptors. Structures of various TIR domains of receptors and adaptors have been determined, and the importance of different structural elements such as the so-called BB-loop has been demonstrated^12^,^13^. The structural basis for TIR-mediated homotypic interactions was first elucidated by the X-ray crystallographic structure of the TIR domain of human TLR2, showing a structural arrangement of five-stranded parallel β-sheets and five surrounding α-helices interconnected by loops^14^. A conserved surface, involving the BB-loop with consensus sequences (F_Y)-(V_L_I)-(P_G) in different Toll receptors and MyD88 homologs, emerges as key to the TIR domain-mediated homotypic protein–protein interaction^15^. MyD88, in particular, is recruited to the activated receptor complex as a dimer that is stabilized by homotypic interactions occurring between death-domain (DD) and TIR domains^15^.

A focal point for the regulation of pro-inflammatory signaling pathways is the exposed BB-loop, which is involved in a dimeric TIR-TIR domain interaction that leads to recruitment and activation of IRAK-1 and IRAK-4 *via* DD. Several structural and mutational studies have pointed to the BB-, DD- and EE-loop regions as mediators of the homo- or hetero-dimerization function of TIR domains in bacteria and mammals^13^,^14^,^15^,^16^. While these studies indicate regions that are involved in dimerization, neither the homotypic nor heterotypic interactions between TIR domains of receptors and adaptors are well understood^17^. More recent mutational studies indicate the critical requirement of amino acid residues in the BB-loop of TIR domain that are involved in the immune signaling specificity of adaptors as well as required for protein self-association (MyD88 homodimerization)^18^,^19^. As homodimerization of MyD88 and its interaction with receptors require the TIR domains, this region is currently being targeted to develop drugs that can block MyD88 activation^20^. It has been reported that an inhibitor peptide that contains a sequence from the MyD88 TIR domain binds to the MyD88 monomer and blocks MyD88 homodimerization^21^. In a recent study, the crystal structure of MyD88 TIR domain was determined, and several TIR-TIR domain interfaces were observed in the MyD88 crystal lattice showing existence of an interface centered on residues R196 and D197 from the BB-loop of one TIR domain that may mimic physiological TIR domain associations^22^.

In this study, we used protein-protein docking to model homodimerization of the human MyD88 protein several years before the dimeric structure became available and only the monomeric structure was known. We evaluated the structural determinants for complex formation of the MyD88 protein-protein assembly to find a druggable site on the TIR domain surface of MyD88 amenable to binding small molecules using PDB entries 2JS7 and 2Z5V^23^ for developing a structural model of the MyD88 dimer. We performed computational docking of over five million commercially-available compounds from the drug-like subset of the ZINC database^24^. Only a small set of top-ranked compounds (culled list of *N *= 30) were actually tested in the first biological assay. A chemical similarity search of PubChem^25^ was performed on the most promising hit to identify even more potent drug-like compounds that are capable of interfering with MyD88 signaling *in vivo*.

## Results

### Docking and structural models predicted MyD88-MyD88 homodimeric complex of the TIR domain

**Figure 1:**
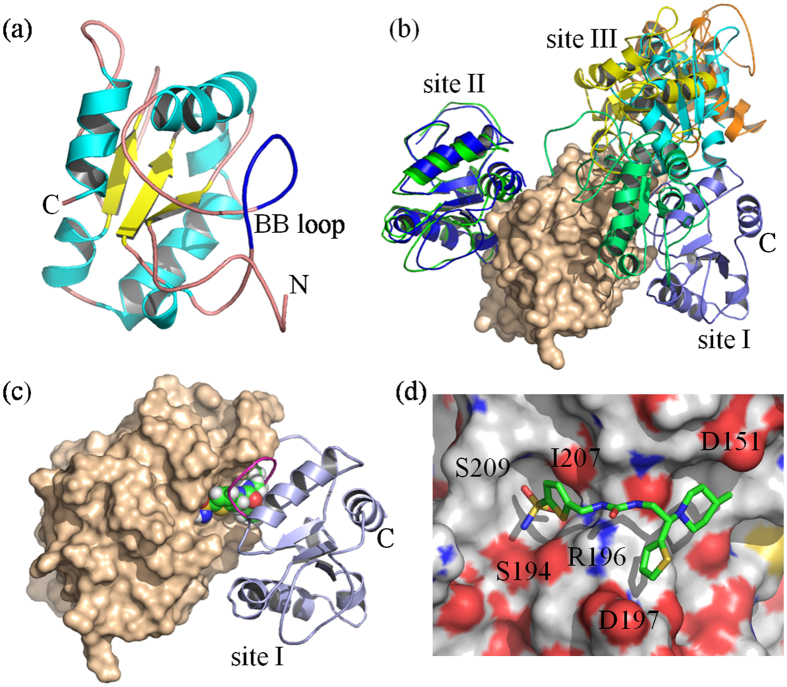
Structural models. (**a**) Conformation of the MyD88 protein fold and the BB loop region (colored dark blue). (**b**) Predicted association sites for MyD88-MyD88 homodimeric complex formation from molecular docking of the TIR domain. The molecular surface represents one MyD88 TIR domain (colored tan). Ribbons diagrams are shown for docked MyD88 structures bound to highly populated association sites observed in SymmDock simulations. The binding site labeled as I is observed to be the top-scoring docked conformational pose. (**c**) Predicted MyD88 TIR dimer of Site I assembly with the BB loop highlighted by magenta color and the small molecule inhibitor illustrated by spheres colored by atom type. (**d**) Conformational pose of the inhibitor T5910047 from *in silico* screening. Surface colors represent polarity of MyD88 atom types and the stick model representation is shown for the docked small molecule ligand.

The MyD88 protein structure from NMR determination^23^ used in the protein-protein docking simulations and screening of the ZINC chemical database for high-affinity ligands is illustrated in Fig. 1. There is a lack of known low molecular weight non-peptide inhibitors of this adaptor protein that bind on the dimeric interface of TIR domain, block dimerization of MyD88, and inhibit downstream signaling. Therefore, we performed a structure-based computational docking screen. The protein fold is a 3-layer αβα sandwich arrangement, and, depending on the specific conformation extracted from the NMR ensemble, the BB loop region is a long coil connecting a short extended segment and a helical structure^23^. The region colored blue in Fig. 1(a) is the residue range of 196–202 and contains the BB-loop sequence motif VLPG. This sequence motif is fairly conserved (25–75%) with other human TLRs, and the proline and glycine appear to be nearly invariant^19^.

Using the displayed MyD88 conformation, Fig. 1(b) illustrates the landscape of sampled protein-protein interfaces from the docking results of SymmDock^26^. Clusters of the most populated association sites are denoted as I, II and III, where site I is the top-scoring dimeric interface from the generated conformational poses. The top-scoring complex found by the symmetry-based SymmDock is observed to be similar to the top-scoring hit from RosettaDock, which performs conformational sampling without geometric constraints^26^,^27^,^28^,^29^,^30^,^31^. In both conformational poses, the BB loop region is at the protein-protein interface and their general agreement provides confidence in the predicted MyD88 complex formation.

The RosettaDock simulations started from a structural alignment of MyD88 onto the pose configuration of the crystallographic structure of the dimer of the human TLR10 TIR domain. Because of conformational differences in the BB loop region, RosettaDock produced a global C_α_ root-mean-square deviation between the starting and top-scoring pose of ~8 Å. This displacement is significant and suggests that conformational selection of either small molecule inhibitors or peptides that mimic the BB loop could conceivably lack universality in their binding poses across the family of TIR-domain proteins.

The binding site I on MyD88, and to a lesser extent site II, can be characterized as a sharp funnel of conformations with strong similarity among the poses, while site III is much more promiscuous in tolerating a broad ensemble of poses. Site I is further categorized by the lowest solvent-accessible surface area of ~15,400 Å^2^ whereas other sites have values that range from ~15,900 Å^2^ for site II and reach as high as 17,240 Å^2^ overall. The funnel shape and interfacial packing of site I are key features for finding optimal inhibitors of MyD88 protein complex formation.

By means of the site I interface of the MyD88-MyD88 contact surface, screening of the ZINC chemical database^24^ was carried out by molecular docking of roughly 5 million drug-like compounds^32^,^33^. Figure 1(c–d) illustrate the complex formation with molecule T5910047 (ZINC12919134)^34^,^35^ which had a LigScore^36^ in the top ten ranking (see Supporting Information for LigScore values of the top screened compounds) and, based on the experimental data presented below, is one of the most promising hits from the first computational screen of 30 compounds tested in biological assays. The binding pose of T5910047 has a recognition signature that is unique among the top-ranked molecules from ZINC and shows a bound molecule that straddles two concave surface elements; one cavity is near residues S194-I207 and is the deeper of the two, while the second cavity is near the charged residue D151. The other top scoring molecules from docking appear to anchor only the S194-I207 pocket region.

**Figure 2:**
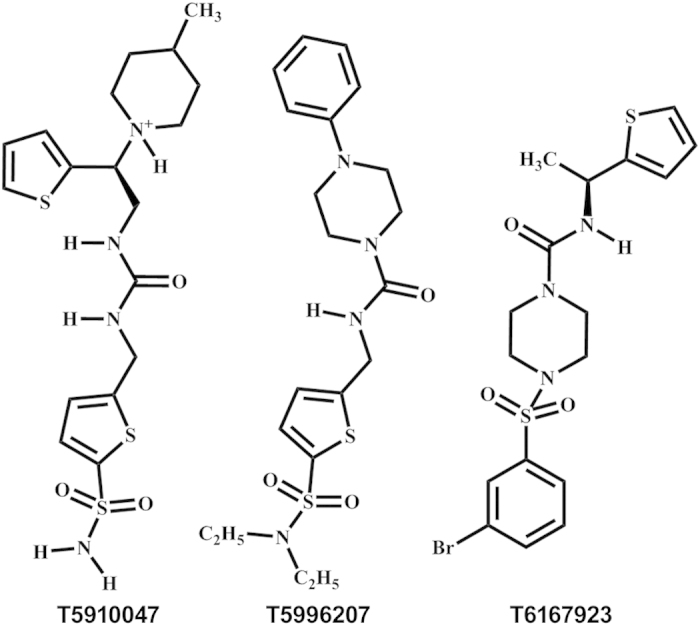
Chemical structures of the 1^st^ round hit (T5910047) and two of the top 2^nd^ generation compounds. The top 2^nd^ generation compounds were obtained by an 80% chemical similarity search of the 1^st^ round hit with a predicted log P < 3.0.

The compound T5910047 was used as a template for a second round search of commercially-available compounds. We queried PubChem^37^ to find molecules that were at least 80% similar to T5910047 and had predicted log*P* values at or below 3.0. Our search yielded ten new compounds that mimicked the proposed binding of T5910047^38^. When assessed in biological assays and *in vivo*, PubChem identified compounds T6167923 and T5996207 (Fig. 2) showed significantly enhanced potency as inhibitors of MyD88-signaling as described below. All three compounds described here were determined to be non-promiscuous via the PubChem Promiscuity server (http://chemutils.florida.scripps.edu:8080/pcpromiscuity/pcpromiscuity.html, see Supporting Information for details.)

### Inhibition of SEB–induced pro-inflammatory cytokine production in primary cultures in the presence of compound T5910047 identified by *in silico* screening

**Figure 3:**
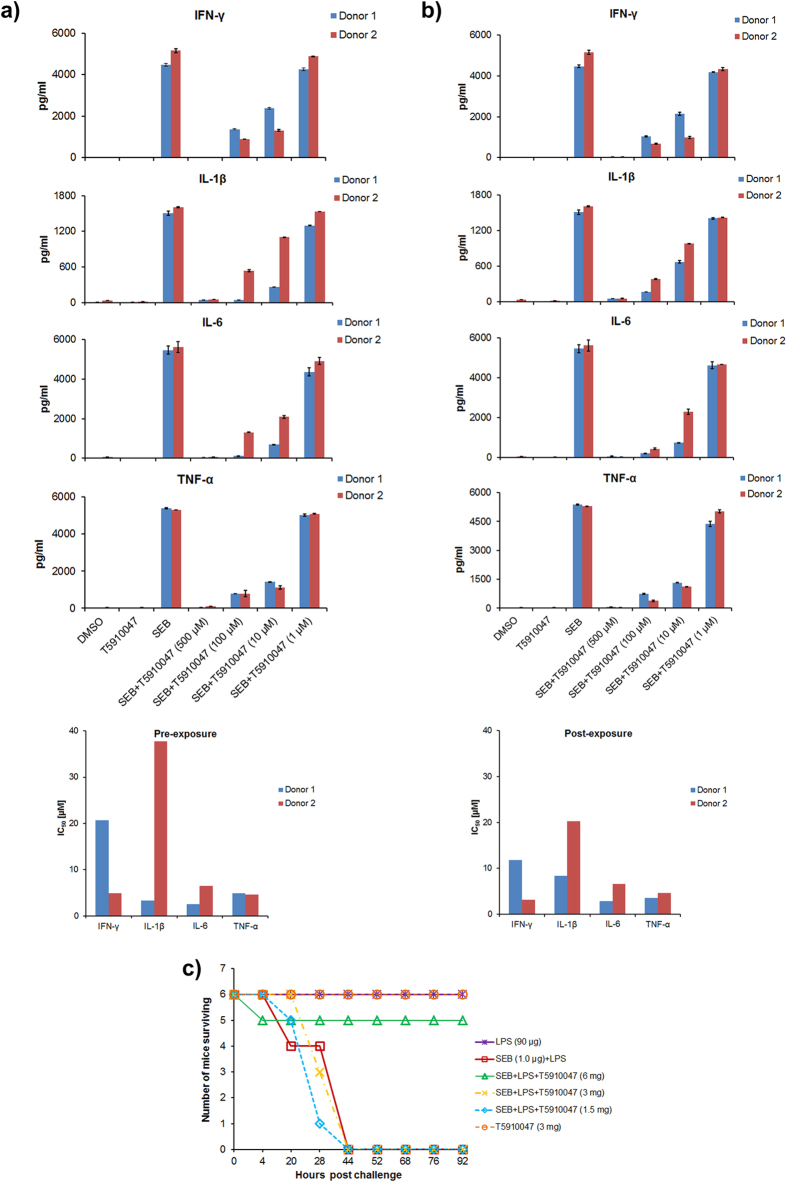
Inhibition of pre and post exposure to SEB -induced cytokine production by T5910047 in primary culture of human PBMC and survivability of mice treated with compound T5910047. PBMCs (1 × 10^6^) from two normal donors were cultured for 20 h with SEB (200 ng/ml) with or without T5910047 (500 μM to 1 μM) treatment 30 min prior to SEB exposure (pre-exposure) or later (post exposure). The culture supernatants were collected and measured for cytokine by MSD assay as described in materials and methods. The data presented as cytokine production (pg/ml) and IC_50,_ calculated as the concentration of the inhibitor required for SEB-induced inhibition of cytokine production in PBMCs by 50% relative to the control (only with SEB treatment without inhibitor). (**a**) IFN-γ, IL-1β, IL-6 and TNF-α production and IC_50_, pre-exposure to SEB. (**b**) IFN-γ, IL-1β, IL-6 and TNF-α production and IC_50_, post-exposure to SEB. Data are the representation of two donors ; (**c**) *In vivo* efficacy of T5910047 - pre-treatment of T5910047 at 6 mg/mouse protected from lethal SEB challenge (P-Values ≤ 0.0131from log-rank test comparing the treated group and untreated group SEB(1.0 μg)+LPS.

To assess the inhibitory effect of compound T5910047 on MyD88-mediated pro-inflammatory cytokine production in primary human cells, peripheral blood mono nuclear cells (PBMCs) were first treated with compound T5910047 for 30 min (pre-exposure) and 30 min after SEB exposure (post exposure). The cultures were incubated for 16 h and culture supernatants were collected to measure cytokine production. While some inhibitory effect of cytokine production was observed with certain inhibitors, the release of most of the pro-inflammatory cytokines such as IL-1β, IL-6 TNF-α and INF-γ in culture supernatant was inhibited in the presence of the T5910047 in a dose-dependent manner (Fig. 3a). The average inhibitory concentration (IC_50_) of T5910047 was found to be in the range of 2–40 μM for most of the cytokines of the two donors tested with some variation between the donors. It may well be that certain cell types are more effectively inhibited than others with different activation kinetics, and certainly with donor-to-donor variation, which is not unusual. Post-exposure to SEB (30 min), T5910047 treatment also inhibited cytokine production (IC_50 _= 3–20 μM) for most of the pro-inflammatory cytokines (Fig. 3b). Consistent with suppressed cytokine production, administration of the compound T5910047 (6 mg/mouse, *p* values ≤ 0.0131) protected mice from toxic shock-induced death with lethal SEB challenge (Fig. 3c). Besides SEB, compound T5910047 also inhibited cytokine production when mouse spleen cells were cultured with *Staphylococcus* enterotoxin A (SEA), lipo-polysaccharide (LPS) or SEA+LPS (Supplementary Fig. S1). To rule out the possibility that some non-specific or MyD88-independent pathways are activated, we performed experiment to determine the MyD88-specific effects are not activated when other than LPS ligand poly I: C (TLR3-ligand) was used. In this ligand induced cell-based reporter assay, MyD88-specific signaling inhibition was determined by SEAP reporter expression in the presence of T5910047 (concentration 500 μM to 0.1 μM). The results indicate a dose-dependent inhibition of MyD88-mediated signaling of SEAP reporter gene expression activated by LPS but not by poly I: C (Supplementary Fig. S2a). To determine a specificity control of the inhibitor T5910047 by using a MyD88-independent response, we used stably transfected HEK293-Blue ^TM^ hTLR3 cells which express SEAP reporter gene driven by the transcriptional control of NF-kB. The results showed that while poly I: C stimulation elicited SEAP reporter expression, but in the presence of the MyD88 inhibitors SEAP expression was not inhibited (Supplementary Fig. S2b). We also examined the activation of NF-kB (p50 and p65) with poly I: C stimulation in the presence and absence of the compound T5910047. The results are consistent with the SEAP expression data and suggest that MyD88-independent NF-kB activation with poly I: C stimulation was not inhibited by T5910047 (Supplementary Fig. S2c). These results demonstrate that the compound T5910047 did not interfere with the TLR3-mediated activation of NF-kB. These results suggest that the compound T5910047, identified through a computational screen, penetrates the cell membrane and targets intracellular MyD88-dependent signaling pathways and is capable of inhibiting SEB/SEA/LPS-induced cytokine production. These *ex vivo* cytokine inhibition data are consistent with our earlier reports that demonstrated MyD88–mediated pro-inflammatory cytokine responses upon SEB or SEA exposure in mice and cytokine production was inhibited in MyD88^-/-^ mice leading to resistance to toxic shock^9^,^10^.

### Search for drug-like compounds that are structurally similar to T5910047

**Figure 4:**
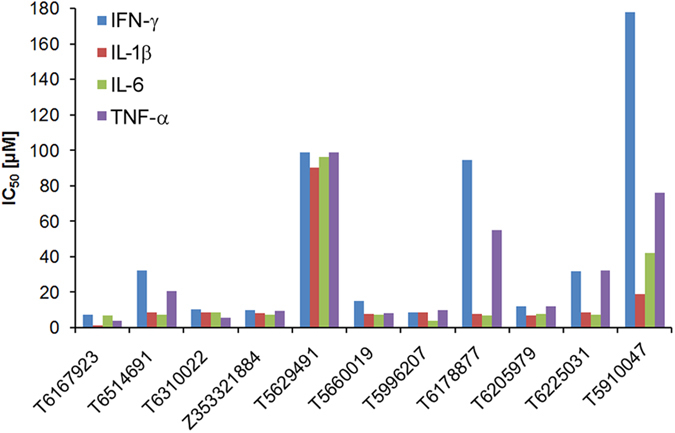
IC_50_ of T5910047 and 2^nd^ generation compounds tested in PBMCs with exposure to SEB. PBMCs (1 × 10^6^) from a normal donor was cultured for 20 h with SEB (200 ng/ml) with or without T5910047 and 10 different 2^nd^ generation compounds identified from PubChem database that were >80% similar to T5910047 using varying concentrations (500 μM to 10 μM). The culture supernatants were collected and measured for cytokine by MSD assay as described in the Experimental section. The data are presented as IC_50_ values, calculated as the concentration required for SEB-induced inhibition of cytokine production in PBMCs by 50% relative to the control. Data represent the IC_50_ values of the inhibitors.

Next, we searched the PubChem database for compounds that were >80% similar to T5910047 but with lower predicted log*P* values^39^,^40^,^41^. Most of these 2^nd^ generation compounds attenuated the pro-inflammatory cytokine response with exposure to SEB in a dose-dependent manner in human primary cells at a much lower concentration than T5910047 (IC_50 _= 2–10 μM) for TNF-α, INF-γ, IL-6, and IL-1β (Fig. 4). The IC_50_ of T5910047 for most of the cytokines was found to be in the range of 2–40 μM when tested with two donors (Fig. 3). Also when the 2^nd^ generation compounds and T5910047 were tested side-by-side in the same donor, the results clearly demonstrated that most of the 2^nd^ generation compounds were indeed better inhibitors of cytokine production than T5910047. An apparent increase in IC_50_ values of the inhibitor T5910047 particularly for the cytokine IFN-γ and TNF-α was noticed in this donor. This is not uncommon to see such a difference of IC_50_ values of an inhibitor from one donor to another donor especially in PBMCs (mixed primary cell populations) which is due to the fact that certain cell types are more effectively inhibited than others with different activation kinetics, and certainly with a difference in activation at the basal level among the donors. For reproducibility of the results as shown in Fig. 4, we tested 2^nd^ generation compounds using PBMCs of a different donor and the results appeared to be consistent (supplementary Table 1 and supplementary Fig. S3).The predicted log*P* octanol/water partition coefficients of the 2^nd^ generation compounds range from 1.2 to 3 (Supplementary Table 2). We selected the most active compounds (T6167923, T5996207) for further characterization because of its lower IC_50_ compared to the other compounds and tested in human primary cultures of PBMCs isolated from multiple donors. The results were similar to Fig. 4, IC_50 _= 2–10 μM (Supplementary Fig. S4a). The compounds showed no toxicity in primary cells up to 100 μM (data not shown). Similar to SEB, the compound T6167923 also inhibited SEA-induced pro-inflammatory cytokine production (Supplementary Fig. S4b).

### Compound T6167923 inhibits MyD88-specific signaling

**Figure 5:**
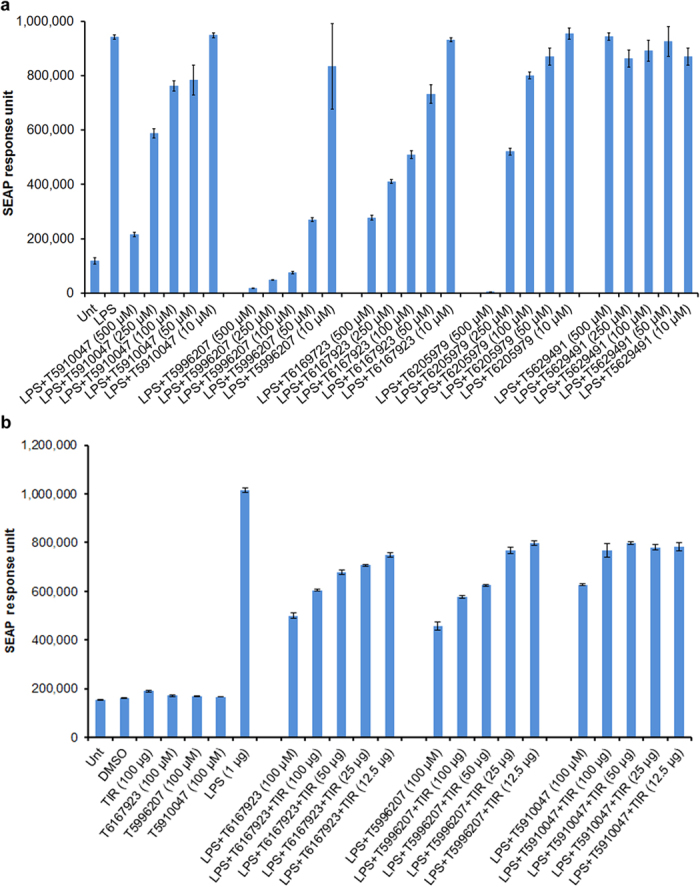
Dose-dependent reduction of secreted alkaline phosphatase response (SEAP) via inhibition of specific MyD88-mediated signaling after LPS stimulation: comparison of original hit and 2^nd^ generation compounds. Compounds were tested by monitoring LPS-induced SEAP activity via a MyD88-mediated NF-kB driven signaling pathway. HEK 293 stable transfected cell line (TLR4-MD2-NF-kB-SEAP) was activated with LPS (TLR4 ligand) and treated with varying concentrations of compounds (500 μM to 10 μM). Culture supernatants were tested for SEAP activity and compared to levels in the absence of compounds. (**a**) Data are presented as SEAP response units. To determine the compounds inhibit MyD88-signaling by direct binding to TIR domain, the compounds T5910047, T6167923 and T5996207 were pre-incubated at room temperature with varying concentration of TIR domain protein with occasional shaking for 2 h and added to cells in a 96 well plates (final volume 200 μl, TIR concentration 100 μg, 50 μg, 25 μg and 12.5 μg and compounds 100 μM). Culture supernatants were tested for SEAP activity and compared to levels of compounds. (**b**) SEAP response unit after pre-incubation with different concentration of TIR domain protein.

To confirm the inhibitory effect of the best compounds on specific MyD88 signaling we tested T6167923, and other compounds, using a ligand-induced cell-based reporter assay. A stable co-transfected (TLR4-MD2-NF-kB/ SEAPorter^TM^) HEK 293T cell line was used to detect LPS ligand-induced MyD88-mediated NF-kB driven SEAP reporter activity. Both the compound T6167923 and T5996207 inhibited LPS induced MyD88 –mediated NF-kB driven SEAP expression in a dose-dependent manner (Fig. 5a). IC_50_ of compound T6167923 and T5996207 (SEAP activity) was in the range of 40–50 μM. To confirm that specific MyD88-mediated signaling, but not MyD88-independent signaling, was inhibited by T6167923 and T5996207, we performed a SEAP reporter assay using (TLR4-MD2-NF-kB/ SEAPorter^TM^) HEK 293 cells after stimulation with LPS (MyD88-dependent) or HEK-Blue^TM^ hTLR3 cells with poly I: C (MyD88-independent) with or without varying concentrations of the inhibitors (500 μM to 0.1 μM, respectively). LPS-induced MyD88-specific signaling of SEAP reporter expression was inhibited in a dose-dependent manner by compounds T5910047, T6167923 and T5996207, whereas, poly I: C (TLR3-ligand) induced SEAP response was not inhibited (Supplementary Fig. S2a, S2b). Consistent with the results of SEAP response, poly I: C induced NF-kB activation was not inhibited (Supplementary Fig. S2c). It is important to note that similar to cytokine inhibition, SEAP activity was inhibited by PubChem identified compounds T6167923 and T5996207 at a lower concentration compared to T5910047. These results suggest MyD88 target specificity of the compounds T6167923 and T5996207 in inhibiting NF-κB driven SEAP activity and lack of effects on MyD88-independent pathways (e.g., TLR3). To demonstrate further that the specificity of the compounds targeting MyD88 by directly binding to TIR domain of MyD88 and functionally reducing MyD88 signaling, we performed LPS-induced cell based SEAP reporter assay after pre-incubation of the compounds T5910047, T6167923 and T5996207 (100 μM) with recombinant TIR domain protein (100 μg, 50 μg, 25 μg and 12.5 μg). Our results demonstrated that a functional reduction of MyD88 signaling with less inhibitory effect of the compounds on SEAP expression in a dose-dependent manner (more SEAP response) after pre-incubation with TIR domain protein compared to without pre-incubation (Fig. 5b). Similarly pre-incubation compounds (100 μM,10 μM, and 1 μM) with 100 μg TIR domain protein showed less SEAP inhibitory activity in a dose-dependent manner compared to without pre-absorption (data not shown). These results clearly demonstrate the specificity of the compounds targeting MyD88 and suggest that direct binding of compounds to TIR protein reduced inhibitory effect of the compounds on MyD88-signaling.

### Compound T6167923 inhibits full-length MyD88 homodimeric formation

**Figure 6:**
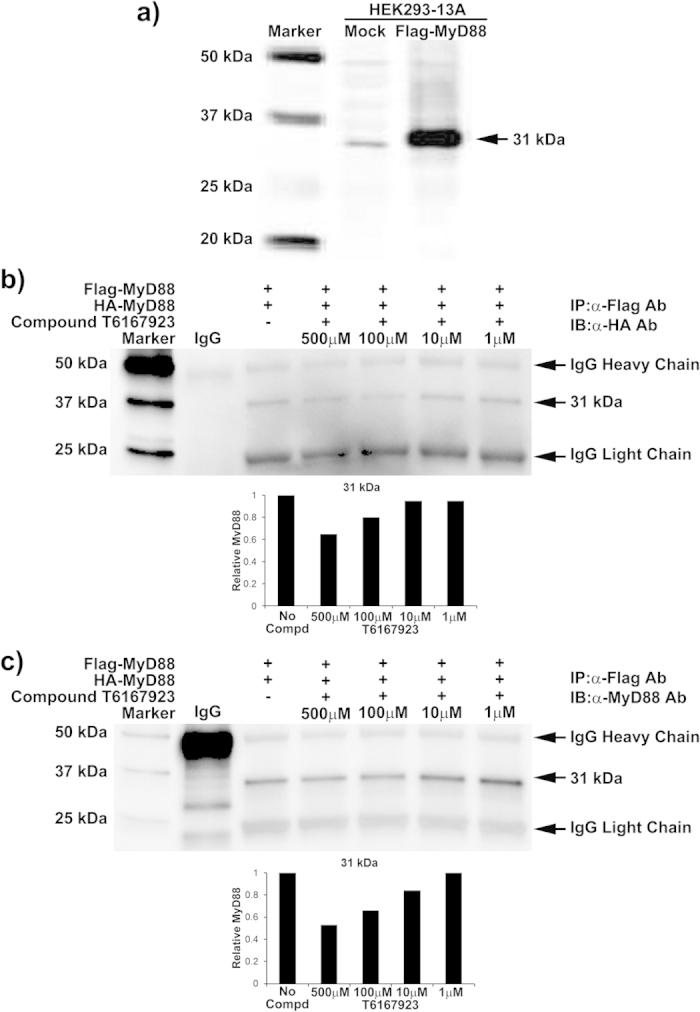
Compound T6167923 inhibited MyD88 dimer formation by targeting newly expressed MyD88. MyD88 knockout cells HEK 293-I3A cells were co-transfected with plasmids MyD88-Flag or pCMV-HA-MyD88. Seven hours after transfection, cells were incubated for 13 h with or without the compounds (500 μM to 1 μM). Cell extracts were immunoprecipitated (IP) with anti-Flag antibody, and immune-precipitated proteins were analyzed by SDS-PAGE followed by Western blotting with anti-HA and anti-MyD88 antibodies. (**a**), MyD88 expression in MyD88 knockout cells HEK 293-I3A. (**b**) Immunoblot probed with anti-HA antibody; (**c**) Immunoblot with probed with anti-MyD88 antibody. Lower panels represent densitometry analyses of the results shown in (**b**) and (**c**). Relative level of MyD88 was determined normalizing the band densities of 31kDa protein in lane 2 (without compound treatment). The results are presented as representative data from two independent experiments.

Our earlier reports demonstrated that SEB stimulation up-regulates MyD88 in primary human cells^7^,^42^,^43^. Having established that T5910047 binds to the TIR domain of MyD88 (Fig. 5), we next asked if T6167293 targets newly synthesized MyD88 when expressed as a full-length protein. For this purpose, we utilized MyD88 knockout cells (HEK 293-I3A) and transfected with plasmid MyD88-Flag. MyD88 expression was confirmed by Western blot analysis (Fig. 6a). Then, we co-transfected HEK 293-I3A cells with plasmids pCMV-HA-MyD88 and MyD88-Flag, and, 6 h later, treated the cells with varying concentrations of compound T6167923. In a co-immunoprecipitation assay using anti-Flag antibody, followed by SDS-PAGE and immunoblot analysis with anti-HA antibody, the 31 kDa MyD88 protein was detected (Fig. 6b). The presence of a 31 kDa band (full-length MyD88) was further confirmed by stripping the gel and reprobing with anti-MyD88 antibody (Fig. 6c). Newly expressed MyD88 was reduced in the presence of T6167923 in a dose-dependent manner. These results suggest that the compound T6167923 targets the newly expressed MyD88 and inhibits dimer formation. In agreement with earlier reports, these results suggest that at higher levels of expression, MyD88 can associate either through its DD or TIR domains^44^,^45^. Thus, compound T6167923 specifically inhibits TIR domain-mediated dimerization of full-length MyD88 as well as the recombinant TIR domain protein (Figs 5 and 6).

### *In vivo* therapeutic efficacy of T6167923

**Figure 7:**
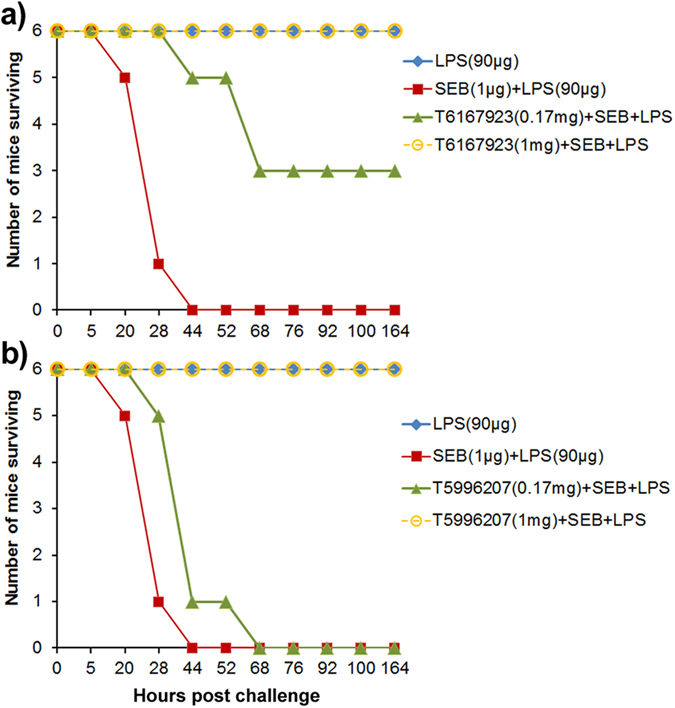
Therapeutic efficacy of the compound T6167923 in mice. BALB/c mice (n = 6/per group) were injected (i. p.) with different amounts of compounds (0.17 mg, or 1 mg), 30 min later injected with 1 μg of SEB (equivalent to 2LD_50_) followed by LPS (90 μg, sub lethal dose) 2 h later. Mice were observed to determine if mice survived, or time of death. Control mice injected only with 90 μg of LPS or 1 μg of SEB survived. (**a**) Survivability of mice with pre-treatment of the compound T6167923 (*P*-values from log-rank test ≤0.0149 and ≤0.0001 of treated group T6167923 (0.17 mg) +SEB+LPS and T6167923 (1 mg) +SEB+LPS, respectively, comparing with untreated group SEB (1.0 μg) + LPS ;(b) Survivability of mice with pretreatment of the compound T5996207. (*P*-values from log-rank test ≤ 0.4592 and ≤0.0001 of treated group T5996207 (0.17 mg) +SEB+LPS and T6167923 (1 mg) +SEB+LPS, respectively, comparing with untreated group SEB (1.0 μg) +LPS. Data represent three separate experiments.

In line with the improved inhibitory effect on pro-inflammatory cytokine production, we checked whether the best 2^nd^ generation compounds could protect an animal from toxic shock with lethal exposure to SEB. To address this question, we used the LPS potentiation model of SEB toxicity in mice as described elsewhere^9^,^10^,^42^,^43^. BALB/c mice (*n *= 6/per group) were injected with (0.17 mg, and 1 mg) amounts of T6167923 or T5996207, and 30 min later they were treated with SEB (1 μg i.e., equivalent to 2 LD_50_), followed by LPS another 2 h later. All animals in the study not treated with the compound died by 44 h. Mice that were treated with T6167923 at concentrations of 0.17 mg had 50% survival (*p* values l ≤ 0.0149) (Fig. 7a). All mice that were treated with T6167923 (*p* values  ≤ 0.0001) or T5996207 (*p* values ≤ 0.0001) at 1 mg were completely protected (Fig. 7a,b). Taken together, the results indicate that T6167923 showed dose-dependent *in vivo* therapeutic efficacy against SEB intoxication.

## Discussion

In this work, we applied a computational screening method to identify selective inhibitors of MyD88-dependent signaling pathways. By screening several million molecules against a structural model of the MyD88-TIR domain homodimeric interface, we identified a small molecule that inhibited SEB-induced MyD88-mediated pro-inflammatory signaling, attenuated cytokine production in primary cultures, and protected mice from toxic shock. Computational docking suggests that this compound binds to a cavity near the solvent exposed BB-loop of the TIR domain interface. In agreement with the computational data, our experimental results suggest that the *in silico* identified compound T5910047 binds to the recombinant TIR domain protein. A chemical similarity search in PubChem yielded several more drug-like compounds with improved activity and therapeutic efficacy in mice.

Recruitment of the MyD88 dimer to the receptor-membrane complex is a requirement for MyD88- mediated signaling *via* activation of the downstream kinases IRAK1 and IRAK4^15^. The TIR domains of the adaptors mediate the initial events that occur after pathogen recognition and thus represent a focal point for the regulation of pro-inflammatory signaling pathways. The specificities of TIR-TIR interactions between adaptors as well as between adaptors and receptors define the formation of various complexes that initiate pro-inflammatory signaling pathways. Structures of different TIR domains from receptors and adaptors have been determined and the importance of different structural elements such as the BB-loop has been demonstrated^12^,^46^. Formation of MyD88 homodimers favors recruitment of IRAK1 into a complex with TRAF6^47^. The MyD88 dimer then dissociates from this complex to be either degraded or reused. Our data from functional SEAP reporter assay detecting the TIR-TIR interaction shows that pre-incubation with the compound with TIR domain protein reduced the SEAP inhibitory effect of the compounds. Thus, the compound T5910047 as well as T6167923 and T5996207 showed TIR domain specificity. Our results demonstrate that the compound binding in this region interferes with TIR-TIR homotypic interactions and thereby inhibits downstream signaling of the pro-inflammatory response. Most of the 2^nd^ generation compounds, including compound T6167923, appear to have log*P* values less than 3 and improved anti-inflammatory activity. Subsequently, our co-transfection experiment with HA- and Flag-tagged MyD88 plasmids in MyD88 knockout cells and immune precipitation data suggest that compound T6167923 targeted the TIR domain of newly expressed MyD88 and prevents MyD88 homodimer formation, which, in-turn, interferes with MyD88 signaling and attenuates pro-inflammatory signaling and toxicity in mice.

## Conclusion

In summary, our structure-based *in silico* approach led to the identification of several small molecule inhibitors of MyD88 that specifically target a cavity near the BB-loop of the TIR domain and inhibit MyD88-mediated pro-inflammatory cytokine production in human and murine primary cells associated with SEA or SEB exposure. The PubChem database search for compounds that were >80% similar to T5910047 but with lower predicted log*P* values^39^,^40^,^41^ led to the identification of several drug-like compounds with improved anti-inflammatory activity. Administration of a single dose of the most effective compound (T6167923) to mice protected them in a dose-dependent manner from toxic shock-induced death following lethal SEB challenge. Collectively, these results provide evidence that small drug-like inhibitors targeting the TIR-domain of MyD88 limit the hyper-inflammatory response, and thus exhibit an anti-inflammatory activity. An ongoing effort is underway to further refine these lead compounds by chemical modification to increase solubility, potency and drug-like properties for potential use against toxic shock and as an anti-inflammatory therapeutic.

## Methods

### Prediction of structural model of the TIR-TIR domain

Structural models of MyD88 were taken from the PDB entries 2JS7 and 2Z5V^23^, both of which are solution NMR determinations. To generate the protein-protein complex of MyD88 and identify a possible binding region for screening drug-like molecules, two rigid-body protein docking simulation methods were applied to 2JS7. For both methods, the most representative conformer of the NMR ensemble (defined by the structure’s authors) was applied. Consensus between docking methods was confirmed for conformational binding poses of the predicted complex assembly.

The first docking method is SymmDock, which is an algorithm developed by Wolfson and co-workers^26^,^27^. SymmDock is a geometry-based docking approach that implements cyclic symmetry constraints of C_*n*_ symmetry on monomeric binding formation. For the work presented here, *n* was set to 2 and the default parameters of SymmDock were applied. Analyzed results consisted of 100 generated conformations of the MyD88 complex and their scoring rank-order.

The second method for protein-protein docking that was applied to MyD88 is the RosettaDock Server of the Gray laboratory^28^,^29^,^30^,^31^. The RosettaDock algorithm works by simultaneous optimization of side-chain conformation and rigid body position of the MyD88 docking partners. The optimization is performed by a packing algorithm and conformational sampling is performed by a rigid-body Monte Carlo minimization strategy. The starting configuration for docking was taken from an structure-structure alignment of the MyD88 structure onto the crystal structure of the monomeric dimer of the human toll-like receptor 10 TIR domain (PDB 2J67). Default parameters of RosettaDock were applied and approximately 980 conformations were scored and analyzed.

### Computational screening methods

The monomeric structure of the MyD88 TIR domain was used for computational docking of small molecules. We used Autodock 4.0^32^ automated with DOVIS 2.0^33^ to screen 5 million drug-like compounds from the ZINC database^24^ against the MyD88-MyD88 binding interface determined from the top-scoring protein docking region. The small-molecule docking box was centered at residue Ser-194 with length of 30 Å. The top 3 structures of the top-scoring 20,000 protein-ligand complexes were minimized with CHARMM^32^ using the MMFF force field^35^ and rescored with LigScore 2.0^36^. Top ranking compounds were visually screened to ensure they overlapped the putative MyD88-MyD88 binding region. A total of 28 compounds were selected for first round experimental testing.

### PubChem chemical similarity search

Using one of the selected small-molecule ligands identified by *in silico* screening (ZINC12919134), enrichment of compounds to be tested experimentally was taken from PubChem (http://pubchem.ncbi.nlm.nih.gov/). Search criteria were set at 90% and 80% structure similarity with final selection based on more favorable X logP3-AA^39^ values that the query input. Final selection of chemical analogs of ZINC12919134 included 5 compounds by the 90% similarity screen and 21 compounds by the 80% similarity screen.

### Reagents

Compounds including T910047, T6167923, and T5996207 were purchased from Enamine, Ltd (La Jolla, CA; see Supporting Information for identity and purity data) and other compounds selected from the computational screen (data not shown) were purchased from ChemDiv, Inc (San Diego, CA). *Staphylococcal* enterotoxin B (SEB) and SEA was purchased from Porton Down, Inc. (Salisbury, UK) and stored at −50 ^o^C. SEA and SEB were endotoxin-free and prepared under GMP conditions. *Escherichia coli* (ssp. 055:B5) lipid polysaccharide (LPS) was purchased from Sigma-Aldrich (St. Louis, MO). Pooled human AB sera were obtained from Pel-Freez (Brown Deer, WI). A cytometric bead array (CBA) kit was purchased from BD Biosciences (San Diego, CA). Meso Scale Discovery (MSD) multi-spot array ultrasensitive cytokine assay kit was purchased from MSD (Gaithersburg, MD). Ficoll-Hypaque was purchased from GE Healthcare Biosciences (Piscataway, NJ). Primary anti-MyD88 antibody was obtained from AnaSpec, Inc. (San Jose, CA). Anti- β-actin antibody was purchased from Cell Signaling Technology (Danvers, MA). HEK 293 (TLR4-MD2-NF-kB-SEAPorter transfected stable cell line was purchased from Imgenex (San Diego, CA). HEK-blue ^TM^ hTLR3 for real –time detection of secreted alkaline phosphatase was purchased from InvivoGen (San Diego, CA). NF-kB p50 and NF-kB p65 detection kit was purchased from Active Motif (Carlsbad, CA). Plasmid 12287 (pCMV-HA-MyD88) and plasmid 13093 (MyD88-Flag) were purchased through an MTA agreement with Addgene (Cambridge, MA). MyD88 KO HEK293 cell line (HEK293-I3A) was a kind gift from G. Stark (Dept. of Molecular Genetics, Lerner Research Institute, Cleveland Clinic, OH). The transfection reagent lipofectamine was purchased from Invitrogen (Carlsbad, CA).

### Mice

Pathogen-free, 16–20 weeks old BALB/c mice were obtained from Charles River (NCI-Frederick, Frederick, MD). All mouse experiments related to therapeutic efficacy test was performed in “accordance” to the approved animal protocol of IACUC.

### Cell isolation and purification

Peripheral blood mononuclear cells (PBMCs) used for this study were obtained from consenting healthy donors in accordance with an Institutional Review Board (IRB)-approved research donor protocol FY05-05. The minimal risk phlebotomy protocol used for human blood drawn from consented volunteers were carried out in “accordance” with the approved guidelines of Office of Human Use and Ethics (OHU & E) . An informed written consent was obtained from all participants and reviewed by the USAMRIID physician (USAMRIID Screening and Eligibility). PBMCs were isolated as indicated in the protocol by standard density gradient centrifugation with Ficoll-Hypaque, harvested from the interface, washed, and suspended in RPMI 1640 medium as described elsewhere^42^. All of the experimental protocols where PBMCs were used for this study “were approved by” OHU&E and Human Use Committee (HUC).

### Cytokine analysis

Cell cultures were incubated (37 ^o^C, 5% CO_2_) for 16 h. Cytokines in culture supernatants were measured by a CBA kit using captured beads coated with antibodies specific for cytokines and flow cytometry analysis as described elsewhere^42^,^43^. Cytokine measurements were confirmed by dilution of culture supernatant using Human Inflammation and Th1/Th2 CBA kits and acquiring 1800 beads. We also used Meso Scale Discovery (MSD) multi-spot array ultrasensitive cytokine assay kit for measuring cytokines in culture supernatants (according to the manufacturer’s protocol). Briefly, the 96-well cytokine assay plate was blocked with diluent 2 (according to manufacturer’s protocol) for 30 min at room temperature with constant shaking at 400  rpm. The calibrators were processed according to manufacturer’s protocol. 25 μl of calibrator and samples were added to the plate in triplicates for 2 h at room temperature with constant shaking at 400 rpm. After the 2 h incubation, the plates were washed 3 times with 1 × PB S+ 0.05% Tween-20, and 25 μl of the detection antibody solution was added to each well of the plate. This was incubated for 2 h at room temperature with constant shaking at 400 rpm. Following the 2 h incubation the plates were washed 3 times with 1 × PBS + 0.05% Tween-20. According to the manufacturer’s protocol, 150 μl of 2 × Read Buffer T was added to each well of the plate and analyzed on the SECTOR Imager. The assay results were read using an MSD SECTOR Image 2400 incorporating a CCD. Sample cytokine concentrations were determined with Softmax Pro Version 4.6 software, using curve fit models (log-log or 4-PL) as suggested by the manufacturer of the specific cytokine. The dose-dependent cytokine production (pg/ml) in the presence of inhibitors was utilized to determine the IC_50_ values from the scatter plot followed by a sigmoidal curve fit using program Origin pro 7.5 as described elsewhere^42^,^43^.

### Secreted alkaline phosphatase (SEAP) assays

TLR4/MD-2/NF-kB/SEAPorter HEK 293 cells (5 × 10^5^ cells/ ml/well) were cultured with LPS (1 μg/ml), or LPS with varying concentration of compounds in 24 well plates and incubated at 37 ^o^C for 16 h. The culture supernatant was collected and centrifuged to remove any cell debris. The Great EscAPe SEAP Assay from Clonetech was used to determine the amount of alkaline phosphatase that is secreted into the supernatant. A 1×dilution buffer is prepared from a 5× stock solution and 75 μl of the 1× dilution buffer is mixed with 25 μl of the supernatant, incubated for 30 min at 65 ^o^C to inactivate endogenous alkaline phosphatase. The samples were then placed on ice for 3 min and then equilibrated at room temperature. SEAP substrate solution (100 μl) was added to each sample and read at 10 minute intervals using a chemiluminescent reader. To determine a specificity control of the MyD88 inhibitor we used HEK-Blue hTLR3 cells. Cells were cultured in DMEM, 10% FBS and supplemented with 30 μg /ml of Blasticidin and 100 μg/ml of Zeocin for real time detection of NF-kB induced SEAP using Quanti-Blue^TM^ by reading the OD at 655 nm according to the manufacturer’s protocol. Briefly, HEK-Blue hTLR3 (1 × 10^6^) cells were suspended in 20 μl PBS were cultured with 180 μl of HEK-Blue detection medium (Invivogen) in a 96 well plate. Cells were stimulated with poly I:C (10 μg/ml) varying concentrations of compounds in the presence and absence of and incubated at 37 ^°^C , in 5% CO_2_ for 16 h. SEAP activity was assessed by reading the OD at 655 nm using a microplate reader.

### NF-kB assay

We used ELISA-based chemiluminescence Trans AM Chemi Kit (Active Motif) and antibodies to detect NF-kB recognition epitope on p50 and p65 in cell extracts of HEK-Blue hTLR3 stimulated with poly I: C in the presence and absence of compounds according to the manufacturer’s protocol (Active Motif). Assays were performed in duplicate to measure NF-kB by using equal amounts of protein (4 μg) from the cell extracts as described elsewhere^7^.

### Expression of MyD88 protein

The open reading frame encoding the TIR domain of human MyD88 (residues 157-296) was amplified from pCDNA3-MyD88-GFP (Addgene plasmid 13026, Addgene, Cambridge, MA, USA) and inserted into pDONR201 (Invitrogen, Carlsbad, CA,). The gene was sequence verified and then inserted into the destination vector pDEST-HisMBP^48^ by Gateway recombinational cloning to generate expression vector pPS2218. The recombinant His-MBP-MyD88 (157-296) fusion protein was expressed in *E. coli* and purified as described^49^,^50^.

### Cell culture and transfections

Human embryonic kidney (HEK) 293T cells were cultured in EMEM, supplemented with 10% fetal bovine serum (FBS) (Invitrogen, Carlsbad, CA), and grown in a 37°C humidified atmosphere of 5% CO_2_. For co-immunoprecipitation of MyD88-Flag/HA-MyD88, HEK cells were cultured in 6-well plates and transfected by lipofectamine 2000 (Invitrogen) method with 4–5 μg of the appropriate plasmids according to the manufacturer’s instructions. The compound T6167923 was added to the medium 6 h after transfection. Six h after transfection, cells were cultured for 24 h and prepared whole cell lysate.

### Co-immunoprecipitation and Western blot analysis

MyD88 knockout HEK 293-I3A cells (transfected or Mock) were collected 48 h after transfection, washed with 2 ml of ice-cold PBS, and lysed in 80 μl of 50 mM HEPES, pH7.4. Cells were pelleted by centrifugation at 10,000 × g for 10 min at 4 °C, and cytosolic fractions were collected for immunoprecipitation. Cell extracts (1 mg total proteins) were incubated with 2 μg of mouse anti–Flag M2 conjugated with agarose attached to magnetic beads (Sigma-Aldrich) for 16 h under constant shaking at 4 °C. Agarose bead-bound immunocomplexes were separated by a magnetic separator, washed three times, and eluted in SDS-PAGE sample buffer and separated by SDS-PAGE and transferred to nitrocellulose membranes. Nitrocellulose membranes were blocked overnight in Tris-buffered saline containing 0.1% Tween 20 and 3% BSA at 4 ^o^C. Blots were extensively washed and probed with anti-MyD88 polyclonal antibody followed by HRP-conjugated secondary Ab and developed with chemiluminescent substrate in the presence of hydrogen peroxide using Immun-Star WesternC Chemiluminescent Kit (BioRad). An imaging system VersaDoc Model 4000 (BioRad) was used to capture the image. The blot was stripped in stripping buffer and then reprobed with anti-HA antibody. Relative level of MyD88 was determined comparing the band intensities.

### Disclosures

Research was conducted under an IACUC approved protocol in compliance with the Animal Welfare Act, PHS policy, and other Federal statutes and regulations relating to animals and experiments involving animals. The facility where this research was conducted is fully accredited by the Association for Assessment and Accreditation of Laboratory Animal Care, International and adheres to principles stated in the guide for the Care and use of Laboratory Animals, National Research council, 2011. Human peripheral blood mononuclear cells used in this study were obtained from healthy donors with written consents, in accordance with guidelines of the human use committee (HUC) and institutional (USAMRIID) review board-approved research donor protocol FY 05-05. Views expressed in this paper are those of the authors and do not purport to reflect official policy of the U.S. Government or USAMRIID/ARL administrators.

## Additional Information

**How to cite this article**: Olson, M. A. *et al*. Discovery of small molecule inhibitors of MyD88-dependent signaling pathways using a computational screen. *Sci. Rep*. **5**, 14246; doi: 10.1038/srep14246 (2015).

## Supplementary Material

Supplementary Information
