# Author Correction: Discovery of small molecule inhibitors of MyD88-dependent signaling pathways using a computational screen

**DOI:** 10.1038/s41598-018-35538-6

**Published:** 2018-11-19

**Authors:** Mark A. Olson, Michael S. Lee, Teri L. Kissner, Shahabuddin Alam, David S. Waugh, Kamal U. Saikh

**Affiliations:** 1 Department of Cell Biology and Biochemistry, U.S. Army Medical Research Institute of Infectious Diseases, Frederick, MD 21702 USA; 2 Department of Immunology, Molecular and Translational Sciences Division, U.S. Army Medical Research Institute of Infectious Diseases, Frederick, MD 21702 USA; 3 Macromolecular Crystallography Laboratory, National Cancer Institute at Frederick, Frederick, MD 21702 USA; 4 Computational Sciences Division, U.S. Army Research Laboratory, Aberdeen Proving Ground, Frederick, MD 21005 USA

Correction to: *Scientific Reports* 10.1038/srep14246, published online 18 September 2015

This Article contains errors.

In Figure 5b, for the experiments with increasing dosage of TIR the labels were listed in the reverse order. The x-axis labels of Figure 5b should read as follows; lane 10 -13 will be LPS + T6167923 + TIR (12.5 µg), LPS + T6167923 + TIR (25 µg), LPS + T6167923 + TIR (50 µg), LPS + T6167923 + TIR (100 µg), lane 16 -19 will be LPS + T5996207 + TIR (12.5 µg), LPS + T5996207 + TIR(25 µg), LPS + T5996207 + TIR (50 µg), LPS + T5996207 + TIR(100 µg) and lane 22-25 LPS + T59910047 + TIR (12.5 µg), LPS + T59910047 + TIR (25 µg), LPS + T59910047 + TIR (50 µg), LPS + T59910047 + TIR (100 µg), respectively. The corrected Figure 5b is published below.

**Figure 5:**
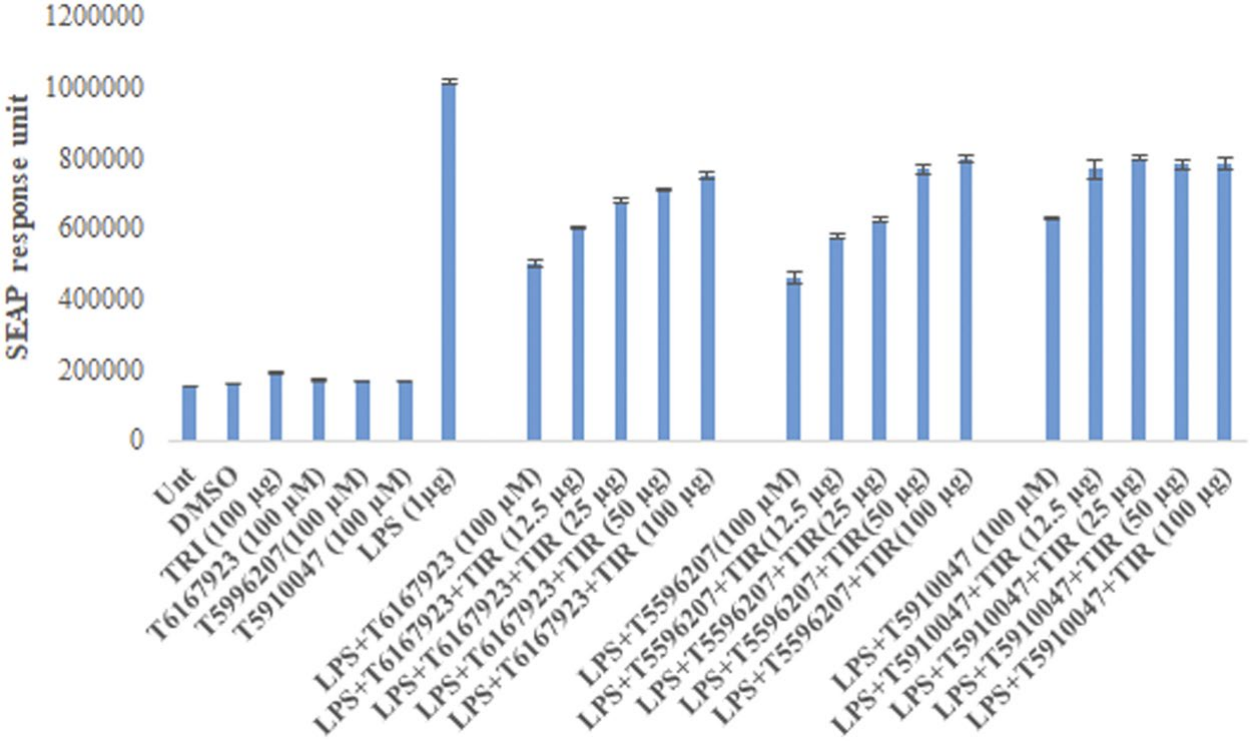
Dose-dependent reduction of secreted alkaline phosphatase response (SEAP) via inhibition of specific MyD88-mediated signaling after LPS stimulation: comparison of original hit and 2^nd^ generation compounds. Compounds were tested by monitoring LPS-induced SEAP activity via a MyD88-mediated NF-kB driven signaling pathway. HEK 293 stable transfected cell line (TLR4-MD2-NF-kB-SEAP) was activated with LPS (TLR4 ligand) and treated with varying concentrations of compounds (500 μM to 10 μM). Culture supernatants were tested for SEAP activity and compared to levels in the absence of compounds. (**a**) Data are presented as SEAP response units. To determine the compounds inhibit MyD88-signaling by direct binding to TIR domain, the compounds T5910047, T6167923 and T5996207 were pre-incubated at room temperature with varying concentration of TIR domain protein with occasional shaking for 2 h and added to cells in a 96 well plates (final volume 200 μl, TIR concentration 100 μg, 50 μg, 25 μg and 12.5 μg and compounds 100 μM). Culture supernatants were tested for SEAP activity and compared to levels of compounds. (**b**) SEAP response unit after pre-incubation with different concentration of TIR domain protein.

This change does not affect the conclusions of the Article.

