# Supplementary material for: Discovery of small molecule inhibitors of MyD88-dependent signaling pathways using a computational screen
Source: Sci Rep. 2015 Sep 18;5:14246. doi: 10.1038/srep14246 (PMC4585646; doi:10.1038/srep14246)
Supplement: Supplementary Information [file srep14246-s1.pdf]

SUPPLEMENTARY INFORMATION

Discovery of small molecule inhibitors of MyD88-dependent signaling pathways using a computational screen

Mark A. Olson[1], Michael S. Lee[1, 4], Teri L. Kissner[2], Shahabuddin Alam[2], David S. Waugh [3], and Kamal U. Saikh[2*]

[1]Department of Cell Biology and Biochemistry, and [2]Department of Immunology, Molecular and Translational Sciences Division, U.S. Army Medical Research Institute of Infectious Diseases, Frederick, MD 21702; [3]Macromolecular Crystallography Laboratory, National Cancer Institute at Frederick, Frederick, MD 21702; [4]Computational Sciences Division, U.S. Army Research Laboratory, Aberdeen Proving Ground, MD 21005

[*] Correspondence and requests for materials should be addressed to K.U.S.

(kamal.u.saikh.civ@mail.mil) or M.A.O. (molson@compbiophys.org)

Tel: (301) 619-4807; Fax: (301) 619-2348

Supplementary Figures 1-4

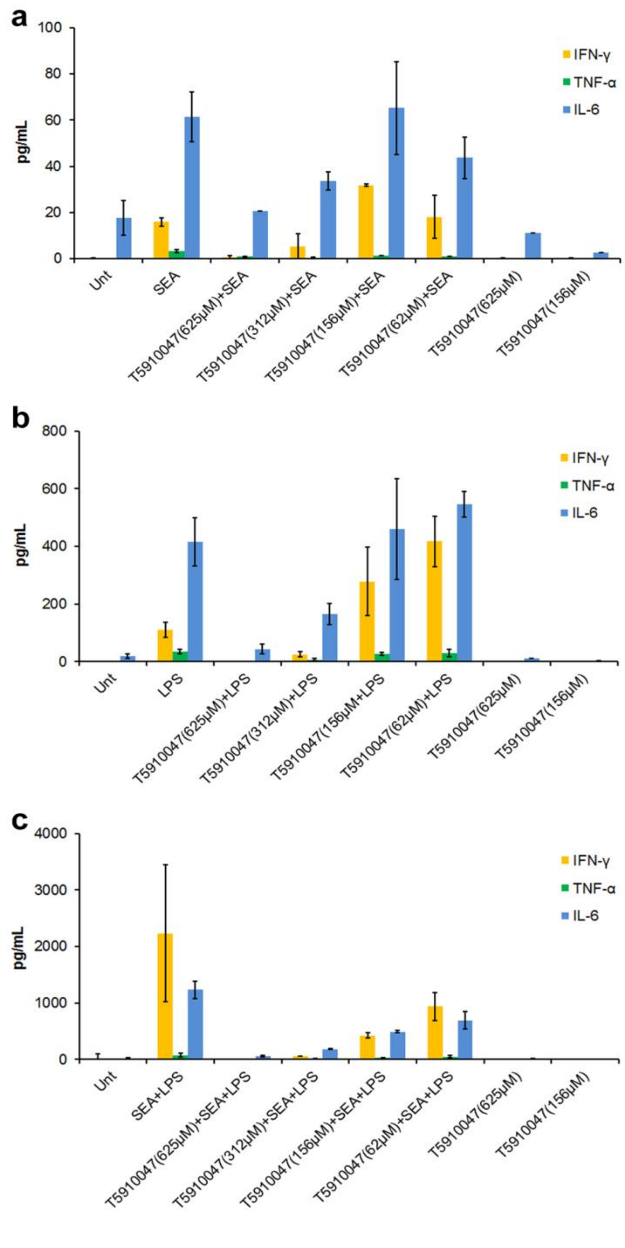

Supplementary Figure 1| Inhibition of SEA -induced cytokine production by T5910047 in primary culture of mouse spleen cells. Spleen cells (1×10^6) from C57/BL6 mice were isolated as described elsewhere[9] and cultured for 20 h with SEA (200 ng/ml), LPS (1µg/ml) or SEA+LPS with or without T5910047 (625 µM to 62 µM) treatment. The culture supernatants were collected and measured for cytokine by CBA assay as described in materials and methods. The data presented as pg/ml cytokine production after exposure to (a) SEA, (b) LPS and (c) SEA+LPS.

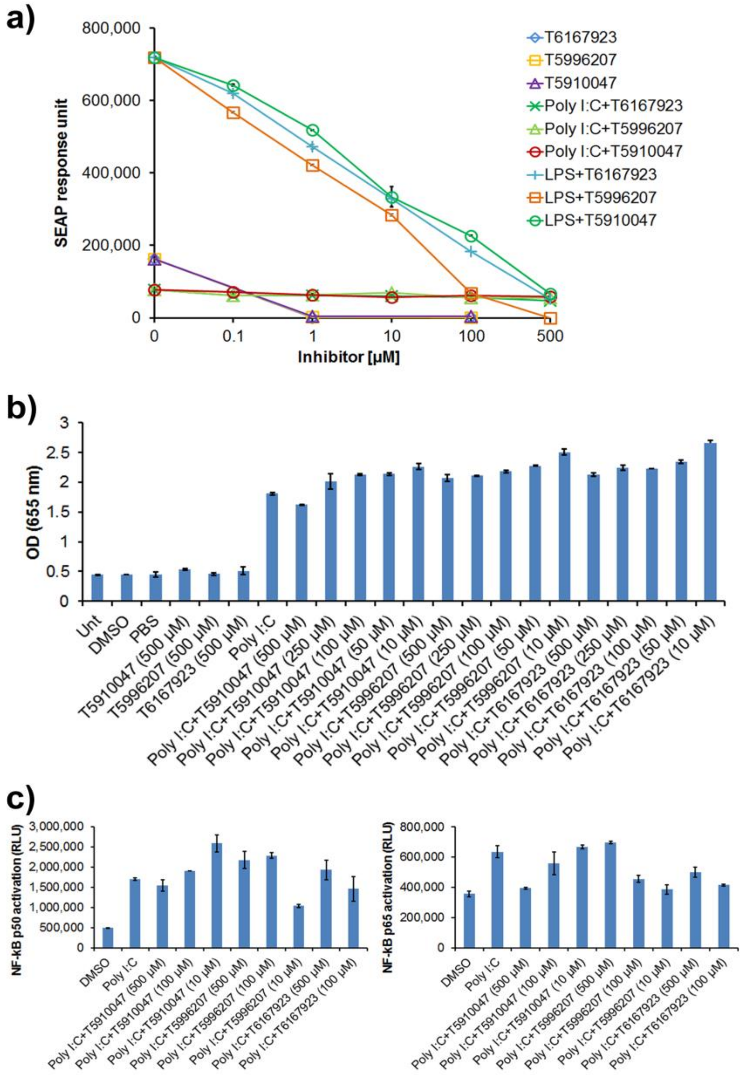

Supplementary Figure 2| Inhibition of MyD88 dependent but not MyD88-independent expression of SEAP activity by compounds T5910047, T6167923 and T5996207. HEK 293 stable transfected cells (TLR4-MD2-NF-kB-SEAP) was stimulated with *E. coli* LPS (1 µg) or poly I: C (10 µg) with or without varying concentrations of the compounds (500 µM to 0.1 µM) respectively. Culture supernatants were tested for SEAP activity. (a) Data presented as SEAP response unit ± SEM. HEK-Blue hTLR3 cells were used to determine a specificity control of the MyD88 inhibitors for real time detection of NF-kB induced SEAP activity after stimulation with poly I:C (10 µg) with or without compound treatment. Real time detection of SEAP response was determined using HEK-Blue detection medium (Invivogen) Quanti-Blue^{TM} by reading the OD at 655 nm. (b) Data presented as OD ± SEM as a measure of SEAP activity. ELISA-based chemiluminescence Trans AM Chemi Kit (Active Motif) and antibodies was used to detect NF-kB activation using NF-kB recognition epitope on p50 and p65 in cell extracts of HEK-Blue hTLR3 cells stimulated with poly I: C in the presence and absence of inhibitors. Assays were performed in duplicate to measure NF-kB by using equal amounts of protein (4 µg) from the cell extracts as described elsewhere [7]. (C) Data presented as a relative luminescence unit (RLU) as a

measure of NF-kB activation of p50 and p65 subunit, quantified using luminescence measurement.

Table1| 2nd generation compounds (PubChem database) were tested at varying concentrations (125 µM, 50 µM and5 µM) in primary culture of human PBMCs exposed to SEB and measured cytokines in culture supernatants by MSD assay (raw data).

Row Labels	Samples	IFN-γ (Human)	IL-1β (Human)	IL-6 (Human)	TNF-α (Human)
U001	MNC untreated	8.441147438	3.5316405	42.306131	16.1557501
U002	SEB 200ng/ml	25615.63251	84.6270145	1182.40974	1668.21251
U003	T6167923 125uM+SEB	1.443718736	1.01513611	4.18217219	3.78850359
U004	T6167923 50uM+SEB	3.36166501	12.9135373	5.71455738	5.05759292
U005	T6167923 5uM+SEB	1953.060442	11.7521809	73.6119476	105.005751
U006	T6514691 125uM+SEB	3.533815999	7.743937	4.73761343	5.25897654
U007	T6514691 50uM+SEB	5.886078258	19.5638474	5.80708099	8.62322433
U008	T6514691 5uM+SEB	14232.68705	32.3457784	106.434784	931.118121
U009	T6310022 125uM+SEB	11.63300254	3.09999687	7.23135844	7.68623827
U010	T6310022 50uM+SEB	12.19498748	18.5772413	7.49750155	7.81141687
U011	T6310022 5uM+SEB	3617.139871	37.9675618	38.0207706	352.054254
U012	Z353321884 125uM+SEB	1.655801913	0.84900824	4.29985022	3.83140695
U013	Z353321884 50uM+SEB	5.479636441	11.0126331	4.98672411	5.36484586
U014	Z353321884 5uM+SEB	10571.45086	28.7827839	127.176008	890.382128
U015	T5629491 125uM+SEB	5.445722573	1.71101766	4.71422181	5.53406729
U016	T5629491 50uM+SEB	8.708475834	10.7268754	6.07647104	7.19517347
U017	T5629491 5uM+SEB	18222.73298	41.1688757	327.787491	1228.44191
U018	T5660019 125uM+SEB	9.375639426	5.08710863	6.42184428	6.25107426
U019	T5660019 50uM+SEB	4.288186253	12.1986029	5.14981855	5.14242363
U020	T5660019 5uM+SEB	8709.595853	34.5664821	431.441831	720.224899
U021	T5996207 125uM+SEB	0.800411598	1.49680879	3.84376488	3.16413095
U022	T5996207 50uM+SEB	2.808746729	12.1648181	4.64400734	4.68578819
U023	T5996207 5uM+SEB	4580.001253	17.3031569	30.7811306	392.564229
U024	T6178877 125uM+SEB	4.799985964	4.28489994	4.9400706	5.65028911
U025	T6178877 50uM+SEB	14.2048218	40.0047542	7.58862709	16.1557501
U026	T6178877 5uM+SEB	8603.732314	52.3935019	314.173684	937.596487
U027	T6205979 125uM+SEB	4.52726201	2.06699318	4.94784791	3.88500969

U028	T6205979 50uM+SEB	5.886078258	12.3201816	5.74540793	4.79213236
U029	T6205979 5uM+SEB	1571.607943	20.1674905	15.974454	245.681198
U030	T6225031 125uM+SEB	0.872551876	0.78520954	3.40841491	3.21813466
U031	T6225031 50uM+SEB	6.729951902	10.0856252	5.65282794	5.76641732
U032	T6225031 5uM+SEB	9886.483591	38.4222049	217.306594	862.546562

Table 2| Characteristics of PubChem identified compounds: Identity (Zn ID), Molecular Weight, % structural similarity with base compound, XLogP3 values and IC_{50} values of compounds calculated from dose response of curve of compounds (raw data as shown in Table 1)

Base compound T 5910047

PUBCHEM Identified Compound ID	ZN ID	Mol. Wt.	% similarity	XLogP3-AA	IC_{50} IFN-γ	IL-1β	IL-6	TNF-α
T6167923	12984105	458	80	2.5	2.7	2.9	2.66	2.66
T6514691	22090889	359	80	1	5.7	4.03	2.75	5.68
T6310022	32889556	492	90	3.3	2.9	4.54	2.58	3.16
Z353321884	26392767	424	90	1.4	4.2	3.79	2.8	5.32
T5629491	22788724	424	80	0.9	6.4	4.9	3.47	11.36
T5660019	9516570	429	90	1.2	3.79	4.23	3.97	4.38
T5996207	9942498	437	80	2.6	2.84	3.12	2.58	3.29
T6178877	26392751	442	90	1.5	3.79	6.57	3.43	5.68
T6205979	32716673	443	90	3	2.66	3.29	2.55	2.94
T6225031	32888206	400	80	1.8	4.1	4.54	3.04	5.2

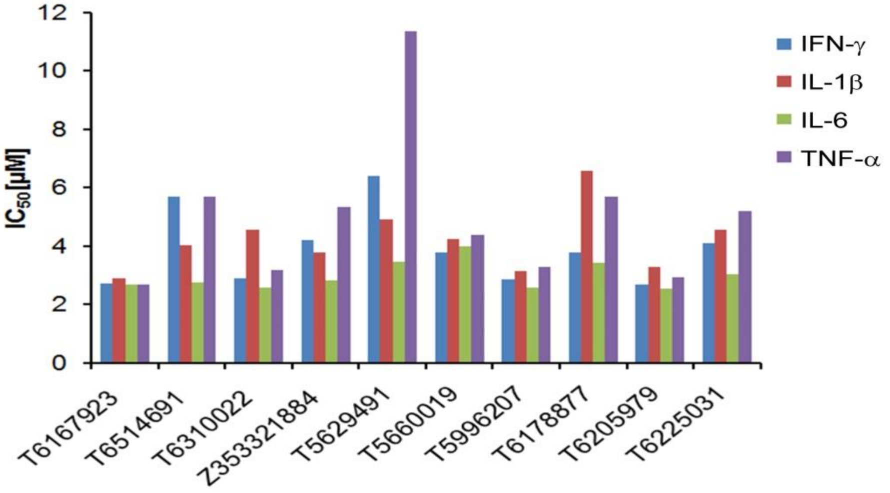

Supplementary Figure 3| IC_{50} of 2nd generation compounds tested in primary cultures of human PBMCs exposed to SEB. PBMCs (1×10^6) from a normal donor was cultured for 20 h with SEB (200 ng/ml) with or without 10 different compounds [(2nd generation) identified from PubChem database that were >80% similar to T5910047] using varying concentrations (125 µM to 5 µM). The culture supernatants were collected and measured for cytokine by MSD assay (**Table 1**) as described in the Experimental section. The compound identity (compound ID and ZN ID), Molecular weight,% structural similarity, and X LogP values and IC_{50} calculated as the concentration required for SEB-induced inhibition of cytokine production in PBMCs by 50% relative to the control are shown in **Table 2**. Data presented as IC_{50}**.**

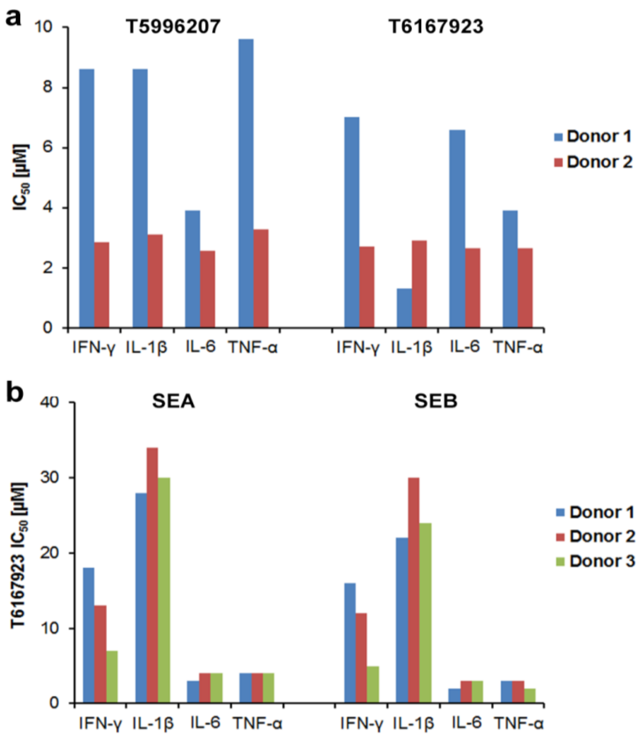

Supplementary Figure 4| Cytokine inhibition by 2nd generation compounds T6167923 and T5996207 tested with exposure to SEB or SEA in primary cultures of human PBMCs isolated from multiple donors. PBMCs (1×10^6) from normal donors was cultured for 20 h with SEB (200 ng/ml) with or without varying concentrations (500 µM to 10 µM) of T6167923 and T5996207. The culture supernatants were collected and measured for cytokine (pg/ml) by MSD assay as described in the experimental section. The data are presented as IC_{50} values, calculated as the concentration required for SEB-induced inhibition of cytokine production in PBMCs by 50% relative to the control.(a) IC_{50} values of T6167923 and T5996207 stimulated with SEB in two different donors;(b) IC_{50} values of T6167923 after stimulation of PBMCs with SEA or SEB in another set of three separate donors.

MyD88 TIR Lig sore sheet

ZINC code - pose # - LigScore2 - # hydrogen bonds

ZINC12645678 2 6.61 3

ZINC12017646 1 6.43 6

ZINC10656259 2 6.42 4

ZINC09741553 1 6.36 3

ZINC09249447 2 6.36 2

ZINC11288001 1 6.35 5

ZINC12919134 **2 6.32 2**

ZINC12697596 1 6.30 4

ZINC12639365 3 6.30 3

ZINC11775323 3 6.29 5

ZINC09598946 1 6.29 3

ZINC09156431 1 6.28 3

ZINC08849913 1 6.28 2

ZINC10989256 3 6.27 4

ZINC10372625 1 6.27 3

ZINC09931075 1 6.27 3

ZINC08718730 1 6.27 2

ZINC02347144 1 6.27 3

ZINC12561210 1 6.26 4

ZINC09733262 2 6.26 3

ZINC09249447 1 6.26 2

ZINC03441998 1 6.26 5

ZINC08707939 1 6.25 1

ZINC12076562 1 6.24 3

ZINC11591799 2 6.24 2

ZINC11448410 1 6.24 2

ZINC12544674 2 6.23 1

ZINC09742768 1 6.23 5

ZINC08923614 1 6.23 2

ZINC09249457 2 6.22 1

ZINC10768353 1 6.21 3

ZINC09727257 1 6.21 2

ZINC09700647 1 6.21 4

ZINC08901921 1 6.21 4

ZINC12641559 1 6.20 4

ZINC10518185 1 6.20 2

ZINC09467273 1 6.20 2

ZINC08763983 1 6.19 1

ZINC07042191 1 6.19 3

ZINC10599911 1 6.18 2

ZINC09779193 1 6.18 0

ZINC10464601 1 6.16 3

ZINC09784908 1 6.16 2

ZINC09249155 1 6.16 3

ZINC12686598 2 6.15 5

ZINC11021637 1 6.15 3

ZINC10967575 1 6.15 1

ZINC10771971 1 6.15 2

ZINC10560140 1 6.15 2

ZINC10458261 1 6.15 1

ZINC10432778 1 6.15 1

ZINC09855102 1 6.15 4

ZINC08820249 1 6.15 2

ZINC09517905 2 6.14 1

ZINC09435421 1 6.14 3

ZINC12109053 2 6.13 4

ZINC11761420 1 6.13 4

ZINC10896492 1 6.13 4

ZINC09576052 1 6.13 4

ZINC08923614 2 6.13 1

ZINC07947566 1 6.13 5

ZINC12889713 2 6.12 2

ZINC11404916 1 6.12 2

ZINC11671915 1 6.11 2

ZINC10349894 1 6.11 3

ZINC09931180 1 6.11 5

ZINC09699354 1 6.11 1

ZINC02206979 1 6.11 3

ZINC12061732 1 6.10 4

ZINC09583421 1 6.10 2

ZINC08800098 1 6.10 3

ZINC11093533 1 6.09 4

ZINC10707080 2 6.09 3

ZINC10087643 1 6.09 2

ZINC09249447 1 6.09 3

ZINC08967022 1 6.09 4

ZINC08806699 1 6.09 3

ZINC12900818 1 6.08 3

ZINC12006544 1 6.08 4

ZINC11358244 2 6.08 2

ZINC11205546 2 6.08 1

ZINC11031418 1 6.08 3

ZINC10874601 1 6.08 2

ZINC10656259 3 6.08 4

23.08.2010

CERTIFICATE of ANALYSIS

1. Identification

Structure	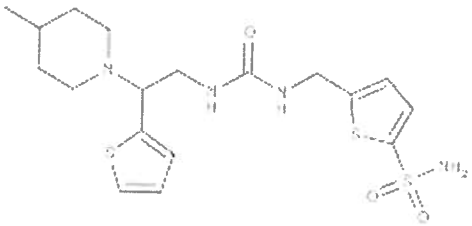
Codes	T5910047
Name	**1-[2-(4-methylpiperidin-1-yl)-2-(thiophen-2-yl)ethyl]-3-[(5-sulfamoylthiophen-2-yl)methyl]urea**
Formula	C18H26N4O3S3
Formula weight	442,61904

2. Description

Appearance	Powder
Colour	brown
Melting point, °C	N/A
Boiling point, °C	Not determined

3. NMR spectra

File name, *wmf	T6410962
Identity	Agrees with the structure

4. LCMS data

File name, *pdf	T6410962
UV area, %	91.82

5. Comments

Comments	No special comments.

ENAMINE Ltd.
23 Alexandra Matrosova Street • 01103 KIEV • Ukraine
Tel.: +380 44 537 32 18 • Fax: +380 44 537 32 53
enamine@enamine.net

T6410962

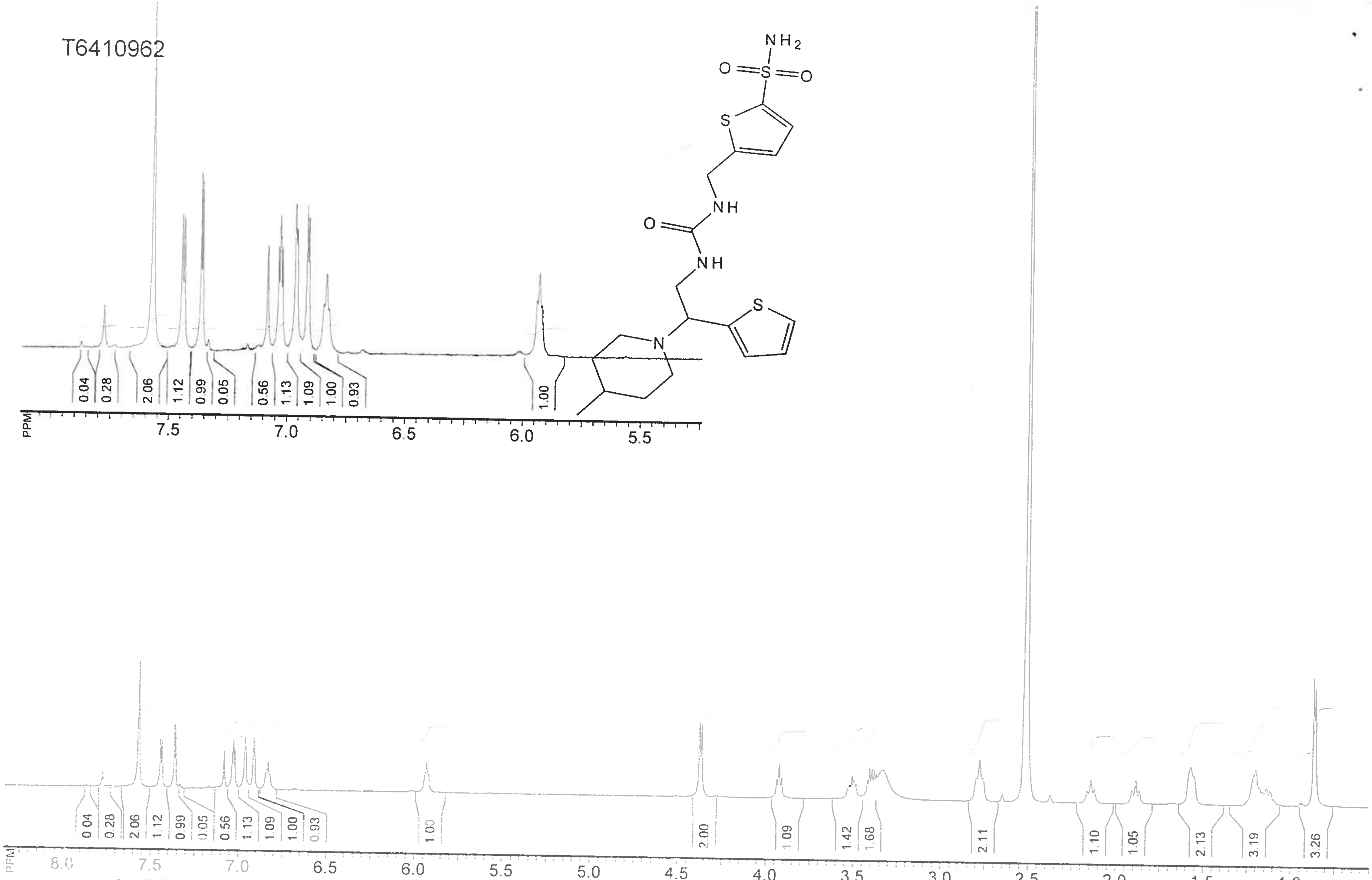

File name: T6410962	Operator: Reutova	SF: 500.0680 MHz	NSC: 1	PW: 0.00 usec, RG: 40	SI: 32768
Date: 16-Aug-2010	Solvent: DMSO	SW: 8013 Hz	TE: 682 K	AQ: 2.04 sec, RD: 0.00 sec	

MaxPeak: 91.82%
Ret_Time: 1.355 min

T6410962

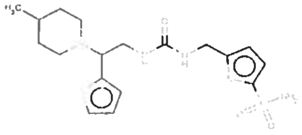

**Mol Wt**	**442.619**
**Exact Mass**	**442.14**

#	Time	Area%
1	1.316	2.06
2	1.355	91.82
3	1.423	4.58
4	1.638	1.54

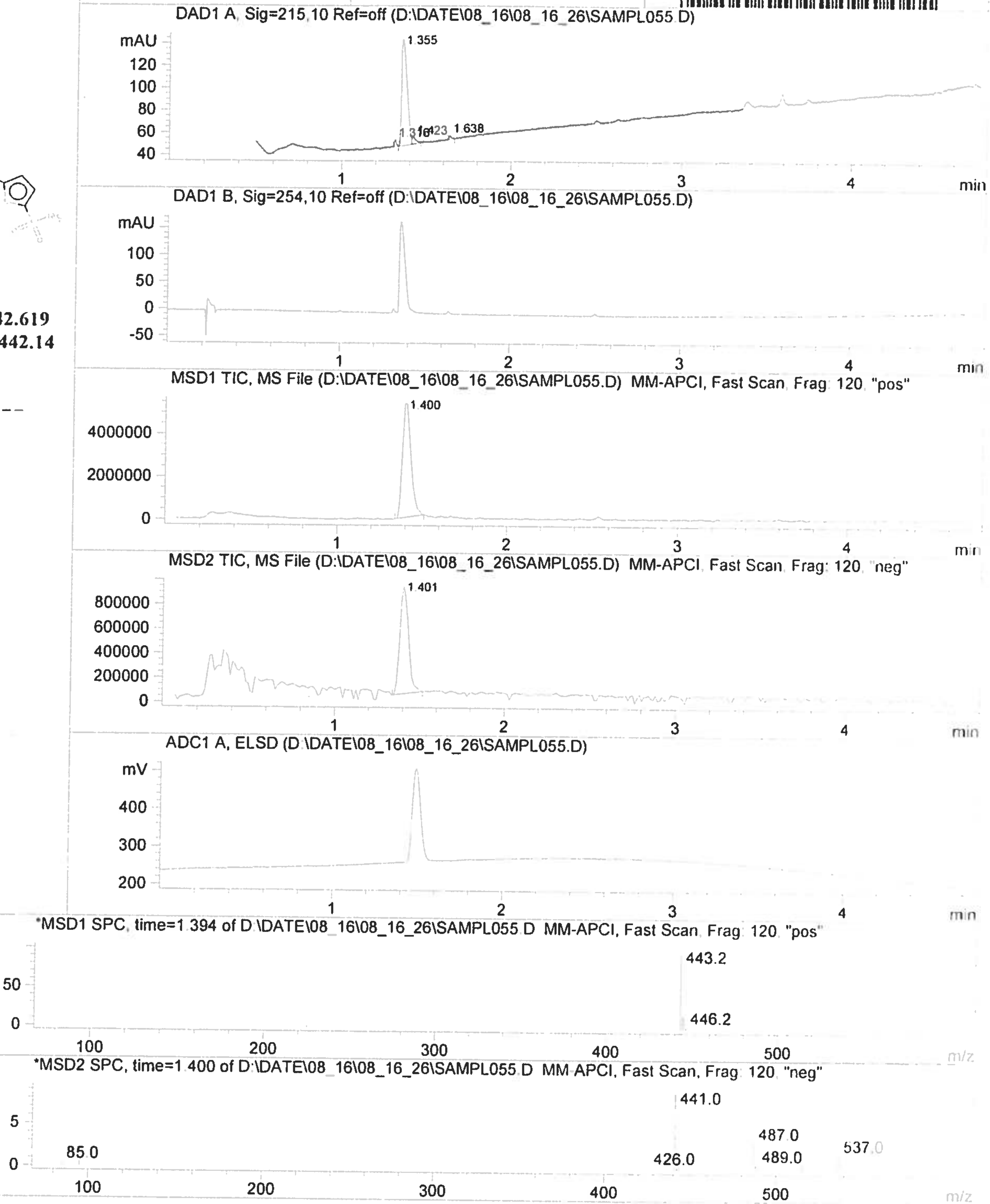

RT 1.400

RT 1.401

Inj.Date 8/17/2010 O P2-F-08 RR Acq. Method C:\CHEM32\-> ->

MATERIAL SAFETY DATA Sheet

1. Product Information

Product Name: **1-[2-(4-methylpiperidin-1-yl)-2-(thiophen-2-yl)ethyl]-3-[(5-sulfamoylthiophen-2-yl)methyl]urea**
Product Catalogue Number: T5910047

Experimental Product for Research&Development Use Only. Not for Drug, Household or Other Use

2. Composition/Information on Ingredients

Product Name: **1-[2-(4-methylpiperidin-1-yl)-2-(thiophen-2-yl)ethyl]-3-[(5-sulfamoylthiophen-2-yl)methyl]urea**
Formula: C18H26N4O3S3
Molecular Weight: 442,61904 AMU

3. Hazards Identification

SPECIAL INDICATION OF HAZARDS TO HUMANS AND THE ENVIRONMENT

Harmful if swallowed or inhalated.

4 - First Aid Measures

AFTER INHALATION
If inhaled, remove to fresh air. If not breathing give artificial respiration. If breathing is difficult, give oxygen.
AFTER SKIN CONTACT
In case of skin contact, flush with copious amounts of water for at least 15 minutes. Remove contaminated clothing and shoes.
Call a physician.
AFTER EYE CONTACT
In case of contact with eyes, flush with copious amounts of water for at least 15 minutes. Assure adequate flushing by separating the eyelids with fingers. Call a physician.
AFTER INGESTION
If swallowed, wash out mouth with water provided person is conscious. Call a physician.

5 - Fire Fighting Measures

EXTINGUISHING MEDIA
Suitable: Water spray. Carbon dioxide, dry chemical powder, or appropriate foam.
SPECIAL RISKS
Specific Hazard(s): Emits toxic fumes under fire conditions.
SPECIAL PROTECTIVE EQUIPMENT FOR FIREFIGHTERS
Wear self-contained breathing apparatus and protective clothing to prevent contact with skin and eyes.

6 - Accidental Release Measures

PERSONAL PRECAUTION PROCEDURES TO BE FOLLOWED IN CASE OF LEAK OR SPILL
Evacuate area.
PROCEDURE(S) OF PERSONAL PRECAUTION(S)
Wear self-contained breathing apparatus, rubber boots, and heavy rubber gloves.
METHODS FOR CLEANING UP
Wipe dry, place a rag in a bag and hold for waste disposal. Avoid fumes inhaling. Ventilate area and wash spill site after material pickup is complete.

ENAMINE Ltd.
23 Alexandra Matrosova Street • 01103 KIEV • Ukraine
Tel.: +380 44 537 32 18 • Fax: +380 44 537 32 53
enamine@enamine.net

7 - Handling and Storage

HANDLING
Directions for Safe Handling: Do not breathe vapor. Avoid contact with eyes, skin, and clothing. Avoid prolonged or repeated exposure.
STORAGE
Conditions of Storage: Keep tightly closed. Keep in room temperature.
Expire date: not available, reanalysis is required no more than once a year.
SPECIAL REQUIREMENTS: -

8 - Exposure Controls / Personal Protection

ENGINEERING CONTROLS
Safety shower and eye bath. Mechanical exhaust required.
GENERAL HYGIENE MEASURES
Wash thoroughly after handling.
PERSONAL PROTECTIVE EQUIPMENT
Respiratory Protection: Government approved respirator.
Hand Protection: Compatible chemical-resistant gloves.
Eye Protection: Chemical safety goggles.

9 - Physical and Chemical Properties

Property Value At Temperature or Pressure

pH	N/A
MP/MP Range	N/A°C
Flash Point	N/A
Flammability	N/A
Autoignition Temp	N/A
Oxidizing Properties	N/A
Explosive Properties	N/A
Explosion Limits	N/A
Vapor Pressure	N/A
SG/Density	N/A
Partition Coefficient	N/A
Viscosity	N/A
Vapor Density	N/A
Saturated Vapor Conc.	N/A
Evaporation Rate	N/A
Bulk Density	N/A
Decomposition Temp.	N/A
Solvent Content	N/A
Water Content	N/A
Surface Tension	N/A
Conductivity	N/A
Miscellaneous Data	N/A
Solubility	N/A

10 - Stability and Reactivity

STABILITY
Stable: Stable.
Conditions of Instability:
Materials to Avoid: Strong oxidizing agents, Strong acids.
HAZARDOUS DECOMPOSITION PRODUCTS
Hazardous Decomposition Products: Carbon monoxide, Carbon dioxide, Nitrogen oxides.

HAZARDOUS POLYMERIZATION
Hazardous Polymerization: Will not occur

11 - Toxicological Information

N/A

12 - Ecological Information

N/A

13 - Disposal Considerations

SUBSTANCE DISPOSAL
Contact a licensed professional waste disposal service to dispose of this material. Dissolve or mix the material with a combustible solvent and burn in a chemical incinerator equipped with an afterburner and scrubber. Observe all federal, state, and local environmental regulations.

ENAMINE Ltd.
23 Alexandra Matrosova Street • 01103 KIEV • Ukraine
Tel.: +380 44 537 32 18 • Fax: +380 44 537 32 53
enamine@enamine.net
